# Modified Two-Stage Approach for the Surgical Treatment of Type II Calcaneal Tuberosity Avulsion “Beak” Fracture: A Case Report

**DOI:** 10.7759/cureus.88416

**Published:** 2025-07-21

**Authors:** Alioune Diane, Daniel Fernandes, Vishwa S Thakor, Amanda Gallagher, Jay Bhuta, Rahul Mittal

**Affiliations:** 1 Orthopedic Surgery, Rutgers Robert Wood Johnson Medical School, New Brunswick, USA; 2 Health Informatics, Rutgers University, New Brunswick, USA; 3 Podiatry, Cooperman Barnabas Medical Center, Livingston, USA

**Keywords:** calcaneal avulsion, calcaneal beak-type fracture, calcaneus fracture, calcaneus tuberosity, orthopedic trauma

## Abstract

Although infrequent, calcaneal tuberosity fractures pose a challenge for surgeons. The treatment goal is to establish stable fixation and restore function of the gastrocnemius-soleus-Achilles tendon complex. Type II fractures, otherwise known as "beak" fractures, are characterized by large avulsion fragments that extend from the posterior calcaneus at the Achilles tendon insertion. Without prompt diagnosis and reduction, these fractures can lead to significant soft tissue damage and severe edema, raising the risk of wound complications and making surgical fixation more difficult. While no widely accepted surgical technique exists for addressing calcaneal tuberosity fractures, clamp-assisted reduction followed by lag screw fixation across the fracture, which may or may not be reinforced by suture anchors, is frequently utilized. However, this approach frequently fails to endure the tremendous pull of the triceps surae and Achilles tendon, leading to failure and calcaneal tuberosity fragmentation. Therefore, there is a need for further research on the optimal method for reducing these types of calcaneal fractures. This report describes a modified two-stage approach used to treat a type II calcaneal tuberosity fracture. Although this treatment required two operations, it has the potential to serve as a good alternative to some of the more common surgical techniques as it negates the Achilles' pull, reduces the fractures, and re-approximates the Achilles.

## Introduction

Calcaneal tuberosity avulsion fractures are rather uncommon but are predominantly seen in elderly women, with a peak incidence in the seventh decade of life. Patients with comorbidities, such as osteoporosis or diabetes, or younger athletes exposed to high-impact activities, are also at an increased risk for all types of fractures [1-3]. The primary cause of most tuberosity fractures of the calcaneus is traumatic stress to the heel induced by sudden, violent contractions of the Achilles tendon resulting from forced dorsiflexion. These fractures are characterized as extra-articular and alongside fractures of the anterior process from the bifurcate ligament and sustentaculum tali, which account for roughly 25% of all calcaneal fractures [4,5]. According to the classification system originally described by Beavis et al., the fracture itself can present as type I, II, or III of calcaneal tuberosity fractures [6]. Type I (sleeve fracture) features a small shell of cortical bone containing the Achilles tendon avulsed from the calcaneal tuberosity [6]. Type II (beak fracture) features an oblique fracture line that runs posteriorly from the most superior portion of the posterior facet [6]. Type III (infrabursal fracture) is a small avulsion fracture that occurs from the middle of the tuberosity [6].

Most cases with significant translation of the posterior tuberosity are treated with an open surgical reduction and internal fixation (ORIF) procedure, along with the use of implants to stabilize the calcaneus. However, there is no established surgical procedure that is standard across all calcaneal tuberosity fractures due to the diverse presentations of the injury. ORIF of the calcaneal tuberosity is associated with profound postoperative risks, including skin necrosis from fracture pressure, soft tissue damage from bone displacement, and calcaneal malunion or nonunion from dislodged implants [7]. A few adapted surgical approaches are currently implemented to mitigate risks associated with an ORIF, including the extensile lateral approach to improve visualization or the sinus tarsi approach to minimize soft tissue damage.

Many alternative methods with altered screw fixation, nail systems, internal fixation plates, suture fixations, and modified ORIF procedures have also been researched to enhance the efficacy of calcaneal fracture reduction [8]. A weakness of most calcaneal tuberosity fracture research cases is that they are limited to small-scale case reports of elderly patients with dissimilar fracture conditions, thereby restricting the generalizability of insights gained from these studies. In this study, we present a technique for repairing a calcaneal tuberosity avulsion fracture utilizing a modified two-stage strategy. The first stage involves pinning a threaded K-wire through the fracture fragment and anchoring it to the superior central calcaneus to serve as a temporary fixation. In the second stage, the Achilles tendon is detached from the calcaneus, followed by ORIF and subsequent repair of the Achilles tendon using the Arthrex Percutaneous Achilles Repair System (PARS) jig (Naples, FL: Arthrex Inc.). This technique aims to negate the pull of the Achilles tendon, preventing the dislodgement of implanted screws, achieving stable fracture fixation, and restoring function of the gastrocnemius-soleus-Achilles tendon complex.

## Case presentation

A 76-year-old female, who takes amlodipine and atorvastatin daily for her history of hypertension and hyperlipidemia, presented to the emergency department at the Cooperman Barnabas Medical Center with complaints of increased swelling in her right foot and ankle and the inability to bear weight on her right foot. The patient reported that she was walking home in the late afternoon after purchasing dinner when she was struck by a car as a pedestrian and fell on her right side. The car drove off, and she walked home, dragging her right leg. She went home and iced the area, believing she had suffered a bad sprain, and reported no additional trauma or loss of consciousness. She denied having any specific pain at home but became alarmed by the progressive swelling in her right foot and called an ambulance to bring her to the emergency department. She reported having only sips of water but denied eating any food or using any other medications, including anticoagulants, and does not have any known food or drug allergies. She is otherwise a healthy woman who regularly follows up with her cardiologist. She was seen by her primary care physician (PCP) one week before this incident for cough and chest congestion, which have since been resolved. She does not report any new changes to her medication regimen.

**Figure 1:**
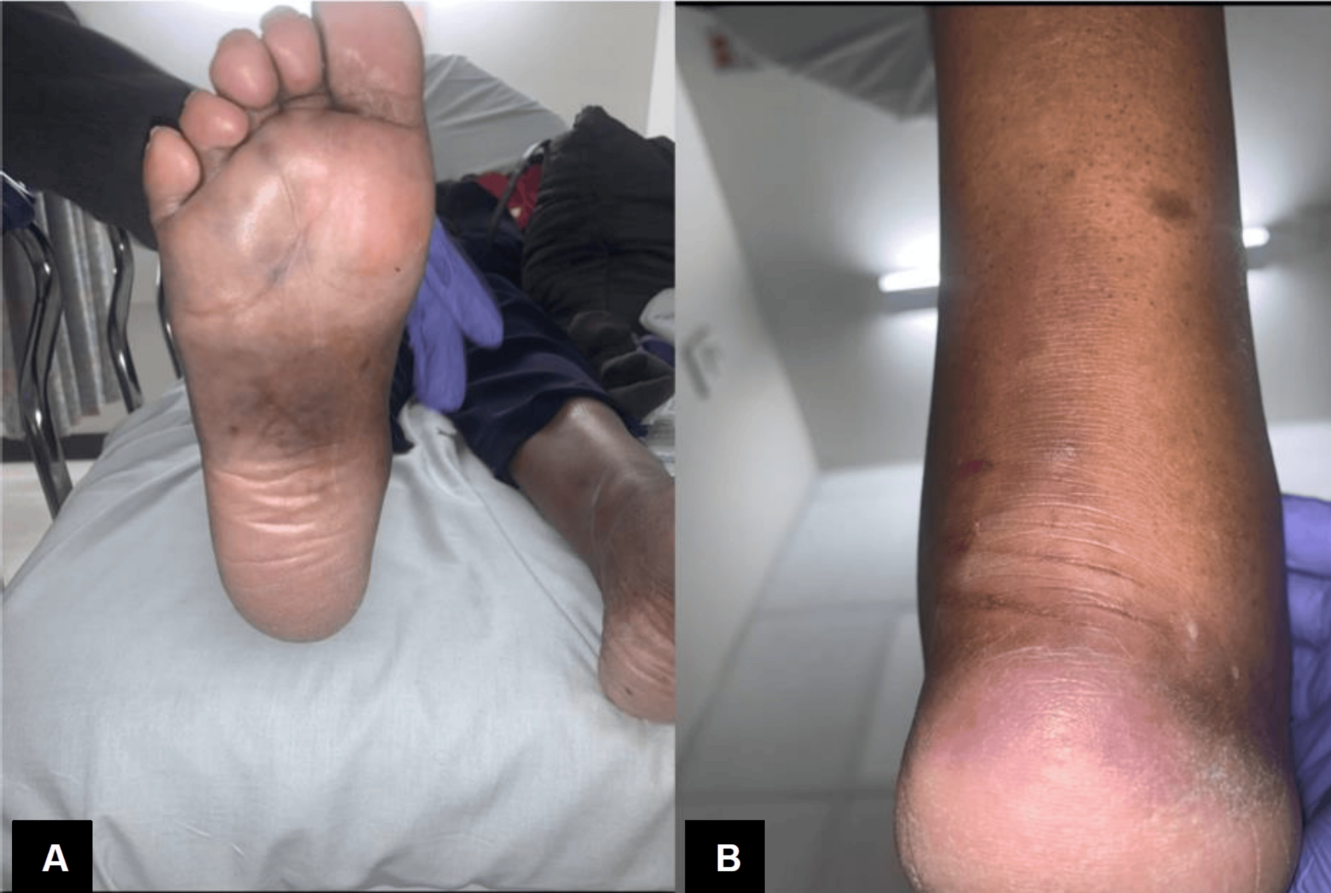
Post-traumatic right foot with Mondor sign. Plantar (A) and posterior (B) views of the right foot displaying ecchymosis, soft tissue swelling, and compromised skin secondary to displacement of the calcaneal tuberosity avulsion fracture.

On inspection, her physical examination was significant for right-sided ecchymosis localized to her foot's dorsal, lateral, and plantar aspect with hematoma extending to the sole of her foot (Mondor sign) and abrasions to her distal posterior leg (Figures 1A, 1B). There was also evidence of significant nonpitting edema to her foot, and skin tenting was noted on her distal posterior leg. She did not exhibit any tenderness to palpation of the foot or with calf compression (Thompson test). Although posterior tibial (PT) pulses were not palpable, dorsalis pedis (DP) pulses were palpable, motor function was present in all digits, and light touch sensation along the foot was intact. The remainder of her physical examination was noncontributory.

**Figure 2:**
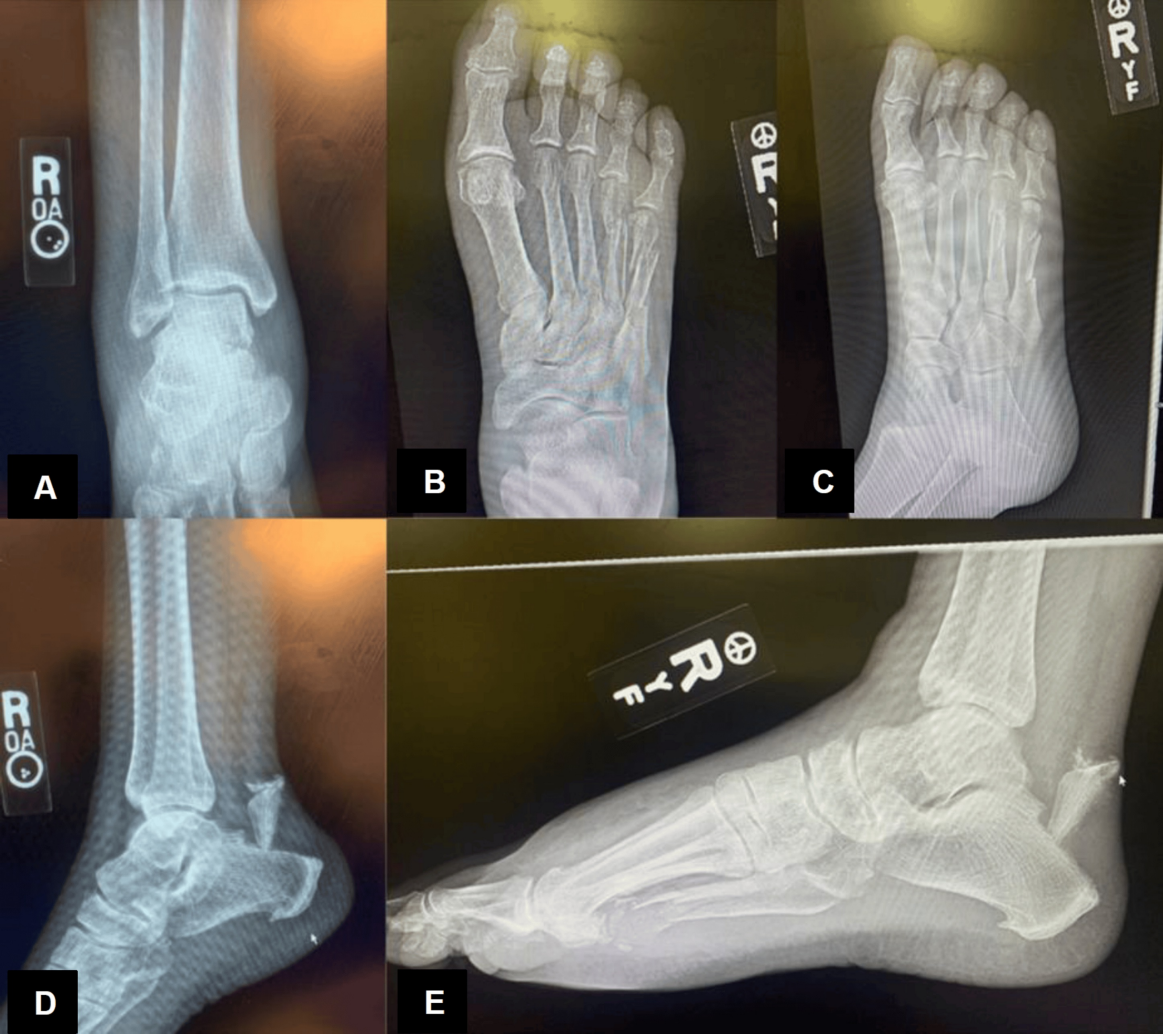
Preoperative radiographs of right foot, ankle, tibia, and fibula. Anteroposterior views of the distal tibia, fibula, ankle mortise, and foot (A and B), medial oblique of the foot (C), and lateral with and without plantar flexion (D and E), radiographs prereduction demonstrating multiple fractures of the second to the fifth metatarsals as well as a large fragment of the posterior calcaneus with 3 cm avulsion.

According to the classification system originally described by Beavis et al., the fracture itself can present as type I, II, or III of calcaneal tuberosity fractures [6]. Radiographs of this patient’s right foot, ankle, tibia and fibula were obtained and demonstrated diffuse osteopenia and soft tissue edema with a large type II fragment fracture of the posterior calcaneus with a 3 cm avulsion, a nondisplaced fracture at the base of the second metatarsal, a comminuted, nondisplaced fracture of the proximal third metatarsal, and lastly a comminuted, slightly displaced fracture of the fourth and fifth metatarsal shafts (Figures 2A-2E).

**Figure 3:**
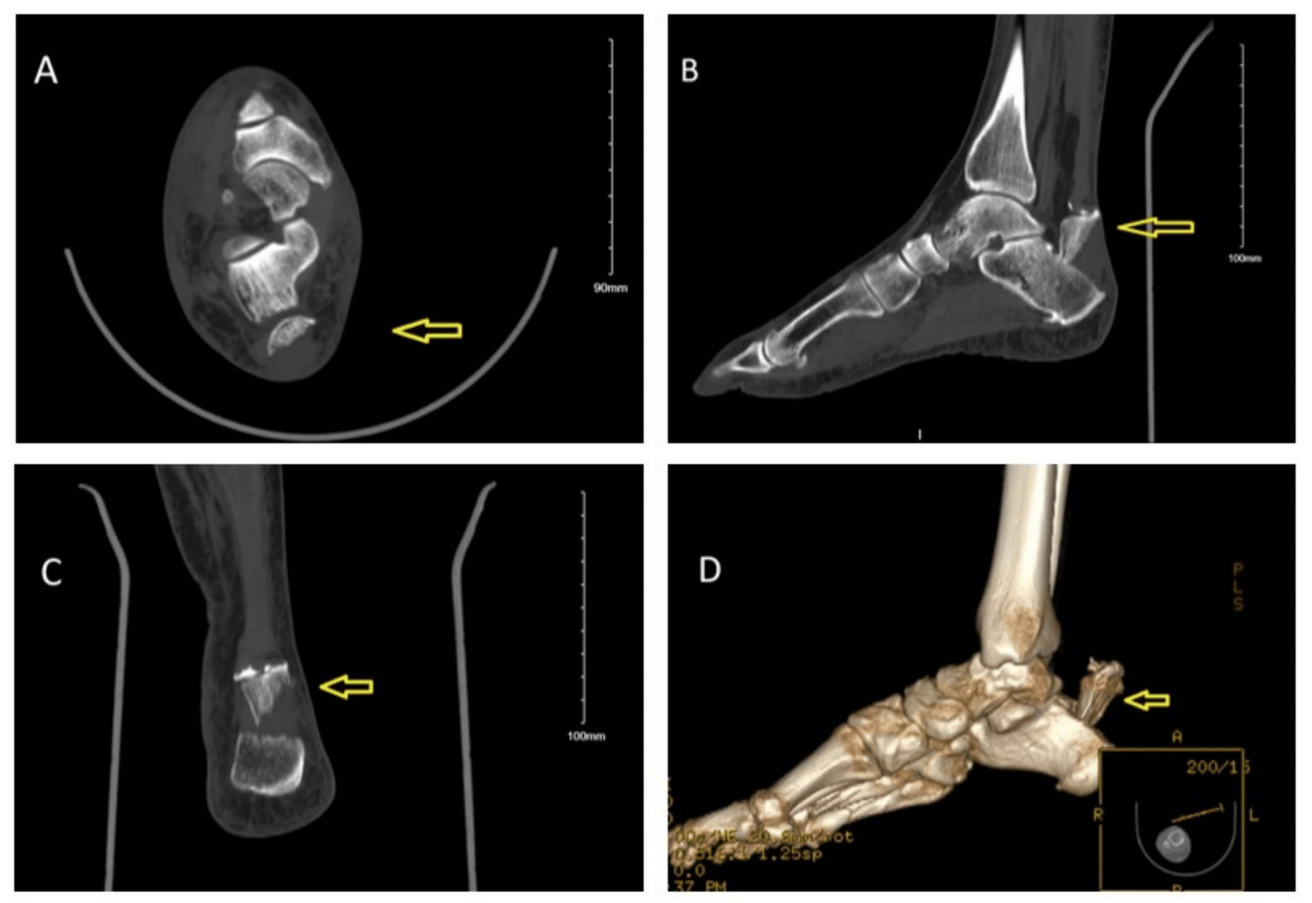
Axial (A), sagittal (B), coronal (C), and 3D reconstruction views of the right foot and ankle. The imaging demonstrates an extra-articular avulsion fracture of the posterior and superior calcaneus (arrows).

To further characterize this patient’s fracture pattern and anatomy, a sagittal and coronal CT reconstructions of her foot was performed and revealed an extra-articular avulsion fracture of the posterior and superior calcaneus that measures up to 3.1 cm in the greatest dimension with associated displacement, and angulation with cranial extension of the Achilles tendon insertion along with smaller comminuted calcaneal fracture fragments and subcutaneous hematoma at the ankle and hindfoot (Figures 3A-3D).

**Figure 4:**
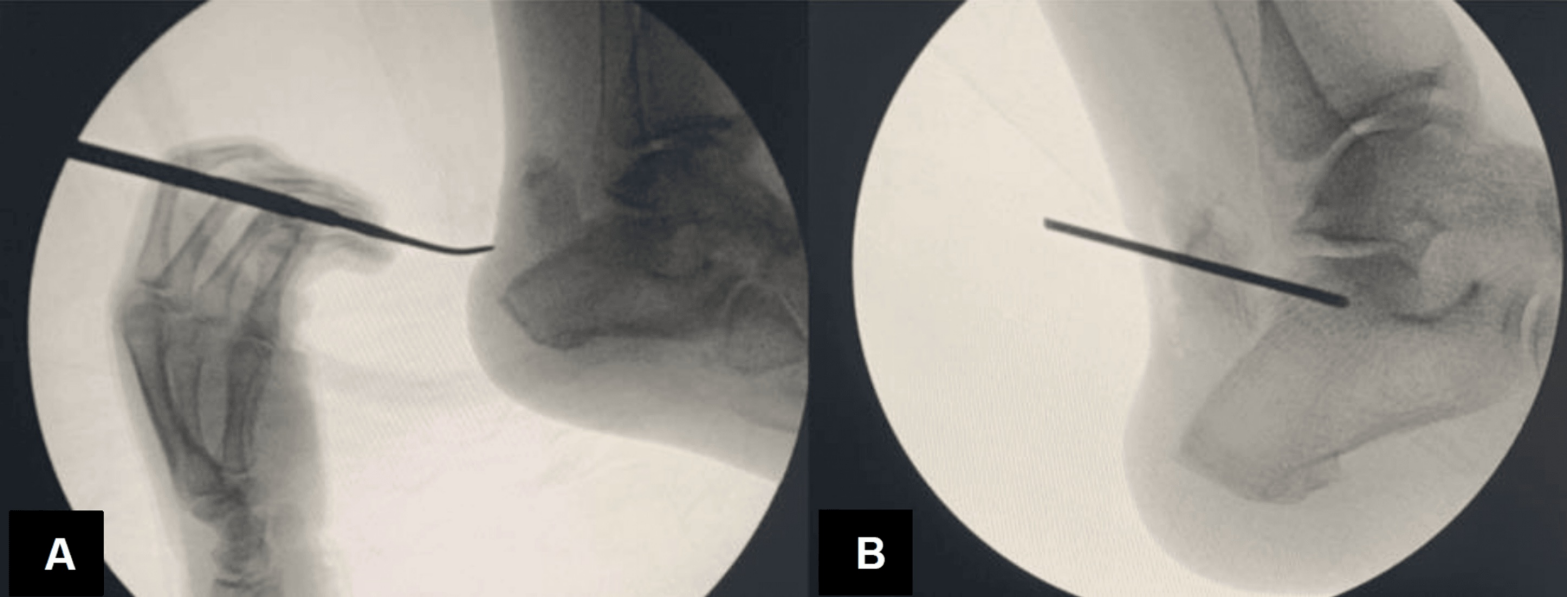
Fluoroscopic radiograph at the level of the calcaneal fracture fragment. Lateral intraoperative radiographs of the right foot immediately before and after initial calcaneal fracture pinning (A and B).

**Figure 5:**
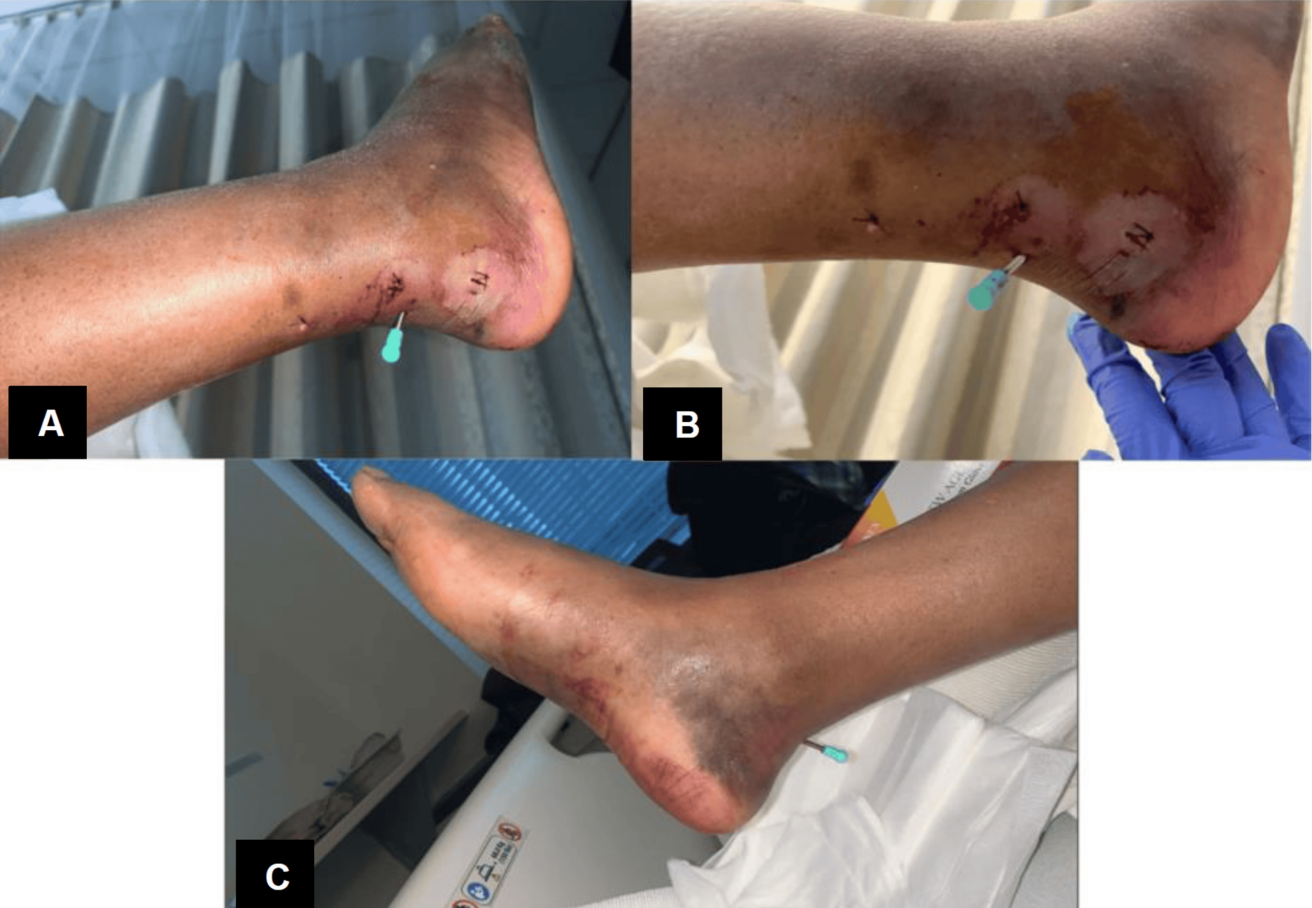
Postoperative day one clinical photos. Posterior and posterolateral views of the hindfoot (A and B) and medial oblique views of the foot (C) are shown in postoperative clinical photos.

The nature of this patient’s injury, along with radiographic findings and physical examination concerning for soft tissue and skin compromise - evidenced by frank skin tenting, nonpitting edema, and overlying ecchymosis and hematoma - deemed this case a surgical emergency to prevent progression to potential skin necrosis, secondary infection, and a limb-threatening situation. She was scheduled for an open reduction internal fixation (ORIF) of her fractured right calcaneus with the podiatry team. However, there was concern about performing a large open incision case to achieve operative fixation, even though the extensive soft tissue swelling was likely to impede the surgeon’s ability to obtain reduction of the fracture using smaller incisions. Using fluoroscopic guidance at the time of surgery, a 1 cm incision was made lateral to the Achilles tendon proximal to the level of skin tenting, and another 1 cm incision was made at the base of the fragment, which was confirmed under fluoroscopy. This was followed by two 1 cm incisions made using a 15-blade, and a hemostat was used for blunt dissection down to bone. A free elevator was used to manually manipulate the large fragment that was noted to still be attached to the Achilles tendon, away from the skin, and mobilized anteriorly. A threaded K-wire was placed through the fragment, anchoring it to the superior central calcaneus (Figures 4A-4B). The incision sites and K-wire site were dressed with Betadine-soaked Adaptic, 4x4 gauze, abdominal (ABD) pad, Kerlix, soft roll, and a posterior splint was applied for immobilization and edema control. On the following postoperative day, the patient’s postoperative dressing was removed to evaluate incision site healing and inspect the surrounding skin for integrity (Figures 5A-5C). Her skin was clean, dry, and intact, and a new dressing was placed.

As complete reduction of the fracture was not possible, secondary to the amount of edema present at the time of surgery, the decision was made to bring this patient back to the operating room eight days later once the swelling was reduced and the skin was no longer compromised to undergo ORIF with removal of hardware and repair of Achilles tendon. Removal was accomplished by first creating a 6 cm longitudinal incision at the posterior aspect of the right ankle just anterolateral to the Achilles tendon to avoid injuring the sural nerve.

Careful, sharp, and blunt dissection were utilized to carry the incision deep through the subcutaneous tissue until the calcaneus, Achilles tendon, and K-wire were all visualized. After the removal of the K-wire, attention was directed to the calcaneus fracture. To separate the avulsed fracture fragment from the Achilles tendon, a full-thickness incision was carefully made just above the periosteal layer of bone. Subsequently, the resultant full-thickness flap was elevated, and a reduction clamp was used to reduce the posterior calcaneus fracture. K-wires were threaded through the fracture fragment, posterior to anterior, to fix the fragment to the posterior calcaneus temporarily. Accurate alignment was confirmed under C-arm fluoroscopy. Once proper alignment was attained, 3.5 mm and 5.5 mm long threaded titanium cancellous screws were drilled through the posterior calcaneal fragment using the AO standard drilling technique, with the K-wires serving as a guide. Positioning was viewed and verified under C-arm fluoroscopy and found to be well-reduced.

To address the remaining void between the Achilles tendon and its insertion point on the calcaneus, the proximal end of the Achilles tendon was grasped using a clamp, and a malleable retractor was used to free the proximal Achilles tendon from the surrounding paratenon. The inner arms of the PARS jig were inserted in the paratenon of the Achilles tendon through the incision and advanced proximally. The suture tails were pulled through the corresponding holes using the needle suture passer. Once all the suture tails were appropriately placed in the tendon, the jig was carefully removed. As per the manufacturer's guidelines, a locking stitch was subsequently incorporated, leaving two transverse sutures and one locked suture. The website PDF guide, as well as multiple surgical technique videos, can be found directly from the Arthrex website [9].

**Figure 6:**
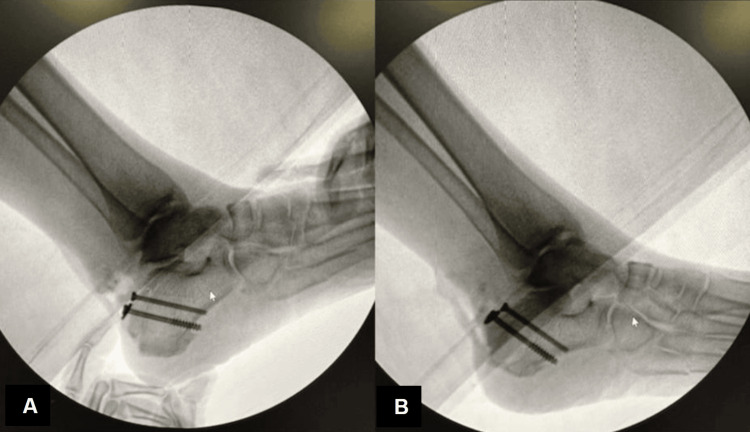
Final radiographs of reduced calcaneal fracture. Intraoperative and immediately postoperative lateral radiographs of the right foot displaying a reduced calcaneal fracture before and after Achilles tendon repair (A and B, respectively).

Attention was then directed towards the distal portion of the Achilles tendon, where two small stab incisions were made over the calcaneus just proximal to the Achilles insertion on the medial and lateral sides of the tendon. A 3.5 mm drill with a 3-0 guide wire was used to make holes at a 45-degree angle converging toward the midline. The drill holes were then tapped, and a Banana Suture Lasso (Naples, FL: Arthrex Inc.) was passed proximally through each stab incision site, and we retrieved the proximal FiberWire strands. These strands were then secured into the calcaneus on the medial and lateral sides with SwiveLock (Naples, FL: Arthrex Inc.) while the foot was held in plantar flexion (Figures 6A, 6B). Given the association between osteoporotic bone and significantly reduced anchor fixation strength, there was some concern that the quality of the anchor fixation strength might not be sufficient to ensure a stable construct. Intraoperatively, we confirmed the adequacy of the repair and strength of the anchor fixation in her osteopenic bone through a negative Thompson test. This demonstrated appropriate plantar flexion of the foot, thus indicating strong anchor stability and intact suture integrity.

The surgical site was then flushed with normal sterile saline. Vancomycin powder was then applied to the surgical site. The surgical site was then closed in layers using 3-0 and 4-0 Vicryl for deep and subcutaneous closure. The skin was reapproximated using 3-0 nylon. The surgical site was then dressed with Betadine-soaked Adaptic, 4x4 gauze, Kerlix, and cast padding up to the knee, and the posterior splint was then applied with the foot in a slightly plantar-flexed position. The tourniquet was deflated at this time after a total of 95 minutes, with immediate reperfusion noted to all digits of the right foot. In the recovery room, she was found to be neurovascularly intact with stable vital signs that were back to baseline.

## Discussion

Calcaneal tuberosity avulsion fractures pose a significant challenge for orthopedic surgeons, accounting for approximately 1-3% of all calcaneal fractures [2]. They are most frequently seen in women in their seventh decade of life, likely secondary to poor bone quality and stiffness of the gastrocnemius-soleus-Achilles tendon complex [2,10,11]. Currently, there is no consensus on the etiology of calcaneal avulsion fractures; however, proposed risk factors include decreased bone mineral density and increased load on the Achilles tendon by the calcaneus, secondary to advanced age, osteoporosis, diabetes, and peripheral neuropathy [12,13].

Anatomically, the calcaneal tuberosity is located in the posterosuperior hindfoot, playing a crucial role in maintaining the arch and serving as the attachment site for the Achilles tendon. In vitro studies have demonstrated that the insertion shape of the Achilles tendon can contribute to avulsion fractures, particularly when the tendon is extensively inserted into the calcaneal tuberosity, thereby increasing the vertical axis of force and propensity for avulsion fractures [7,14,15]. The calcaneus is primarily comprised of cancellous bone with a thin cortical layer, and the density of the trabeculae determines its strength [16]. Moreover, the force exerted by the gastrocnemius-soleus-Achilles tendon complex on the calcaneal tuberosity can support up to three times a person’s body weight during active movement [8].

Calcaneal tuberosity fractures are extra-articular and typically result from forced dorsiflexion, sudden contraction of the gastrocnemius-soleus-Achilles tendon complex with the knee in full extension, or direct trauma [2]. There are three main classification systems to characterize extra-articular fractures as follows: Beavis, Essex-Lopresti, and Sanders. The Beavis classification system categorizes these fractures based on their morphology as follows: type I (sleeve fracture), type II (beak fracture), and type III (infrabursal fracture) [6]. Lee expanded this classification to include type IV, a beak fracture with a small triangular fragment [7]. The Essex-Lopresti classification features the joint depression type, a single fracture line that runs obliquely through the posterior facet separating the anterior and posterior portions of the calcaneus, and the tongue type, which has the same fracture line as a depression type with another horizontal fracture line running posteriorly, creating a superior posterior fragment [5,17]. Lastly, the Sanders classification consists of four different fracture types based on the number of articular fragments viewed on the coronal CT image at the widest point of the posterior facet, and the increasing number of fragments reflects an increased fracture severity (type IV) [4].

In this case, a 76-year-old female sustained a Beavis type II calcaneal tuberosity avulsion fracture following a motor vehicular collision, though she did not have any of the previously mentioned risk factors, such as diabetes or peripheral neuropathy. Type II fractures can cause superior translation of the beak fragment due to traction from the Achilles tendon and lead to skin compression and ischemic necrosis. Early surgical intervention is recommended for type II fractures to avoid posterior skin necrosis. Cast immobilization for 10-12 weeks can be used for tuberosity fractures with less than 1 cm displacement and no threatened soft tissue, particularly in elderly patients with reduced mobility. However, non-surgical management can lead to higher rates of malunion and impaired dorsiflexion, making surgical intervention the preferred option for most cases [10]. This patient exhibited significant ecchymosis, hematoma, nonpitting edema, and skin tenting, indicating a surgical emergency. Moreover, as per the de Soto classification, if the displacement in a type II Beavis is >2 cm, the skin is more likely to rupture [18]. In this case, the patient had a 3 cm avulsion of the calcaneal tuberosity, indicating there was a significant threat of soft tissue compromise.

Surgical approaches to calcaneal tuberosity fractures vary based on fracture type, associated injuries, and surgeon preference. Treating these fractures is exceptionally difficult because the stability of the reduced bone block must be balanced with the traction of the Achilles tendon. Normally, the gastrocnemius-soleus complex produces between 1962 and 2354 N of traction on the Achilles tendon [19]. Thus, an ideal reduction would provide 369 N of stability in neutral dorsiflexion with complete reduction of the bone block, no soft-tissue compromise, and a functioning Achilles tendon [20]. Historically, K-wires and screws have been used for type II fractures, providing strong fixation but posing a risk of soft tissue injury. K-wire tension band constructs, cortical screws, anchor screws, and plate screws have also been utilized, each with their risks and benefits. Type III and IV fractures generally require more robust fixation with suture-bridge techniques, side-locking loop suture techniques, and tight-rope techniques.

The primary goal of surgery is to provide stable fixation that allows restoration of the gastrocnemius-soleus-Achilles tendon complex that can resist the pulling force of these muscles [10]. This case involved a two-stage surgical approach for a type II fracture, initially using a pin-threaded K-wire to temporarily reduce the fracture. The K-wire placement allowed for immediate reduction of skin tenting, which is essential to prevent skin necrosis and vascular compromise. At the time of presentation, the limb was deemed to be too edematous to proceed with an open procedure due to the surgeon’s concern of being unable to adequately close the incisions due to the edematous nature of the soft tissue envelope. In such cases, it is within the standard of care to first relieve any tension on the skin to prevent necrosis and to delay ORIF until the soft tissue envelope has normalized.

Definitive fixation was delayed for eight days to allow for soft tissue healing. The second stage involved using a reduction clamp and two temporary K-wires, followed by inserting two titanium cancellous screws for definitive fixation. The Achilles tendon was re-attached using the PARS technique. The reported downsides of this technique include a relatively increased cost due to specialized instrumentation and suture materials, as well as a low risk of sural nerve injury [21]. However, the authors elected to use the PARS technique because of its ability to provide strong fixation that is biomechanically comparable to open repairs and other minimally invasive methods, while also lowering the overall risk of wound complications and infection via its percutaneous approach, as depicted in the literature [22-24].

In their biomechanical studies, Melcher et al. and Clanton et al. discovered that, despite the possibility of slightly less early elongation under cyclic loading from the suture-bridge constructs, the PARS technique achieved comparable or better ultimate strength and cycles to failure with less soft tissue dissection and a lower risk of wound healing issues [22,23]. Similarly, because of the decreased elongation, the use of the side-locking loop approach also permits more aggressive early rehabilitation [25]. Nevertheless, the side-locking group was associated with a higher risk of sural nerve injury, while the PARS technique demonstrated equal or superior resistance to failure [25,26]. In addition to reports indicating that most patients return to baseline activities within five months of PARS utilization, tight-rope and suture-bridge techniques frequently necessitate more extensive hardware or anchor use, increasing case complexity and cost without a clear advantage over PARS in clinical outcomes [24,27,28].

After conducting an extensive literature search, the authors have not found this particular two-staged approach to fixing type II calcaneal tuberosity avulsion fractures described in the literature. McCarthy et al. describe a technique used for a 59-year-old male with a type II calcaneal tuberosity fracture with simultaneous Achilles tendon rupture in which two 5 mm threaded screws were used for fixation, and the Achilles tendon was re-attached using two Mitek anchors with a modified Bunnell technique [29]. Tanpure et al. describe the use of two corticocancellous screws, with one passed perpendicular to the fracture and another passed obliquely to nullify the force of the Achilles tendon, though the patient was notably a 29-year-old male [30]. Villalba et al. also reported a 72-year-old woman with a Beavis type I posterior calcaneal avulsion fracture and associated Achilles tendon rupture treated initially with a long-leg cast and delayed surgical intervention with cannulated screws for the calcaneal fragment, Krackow suture for the Achilles tendon [31]. Our described surgical technique demonstrates that, especially when there is substantial soft tissue edema, a staged approach is a viable alternative to the traditional single-stage fixation. The method was designed to lessen the Achilles tendon’s tensile forces, lower the possibility of implant displacement, and provide solid fixation while minimizing the soft tissue and vascular damage brought on by skin tenting. Although there are inherent risks to performing two operations, the authors of this study feel it has the potential to address the major challenges associated with type II calcaneal tuberosity fractures.

## Conclusions

Despite the rarity of these type II “beak” fractures of the calcaneal tuberosity, they can be difficult to manage, and the appropriate surgical technique has yet to be standardized. The main objective was to achieve stable fixation and restore the gastrocnemius-soleus-Achilles complex’s functional integrity, regardless of the repair method selected. Timely detection and treatment of these fractures are essential since delayed action may result in progressive edema and soft tissue injury, making surgical intervention more difficult and raising the risk of unfavorable outcomes. To accomplish this, it is critical to design a construct that can resist the tension created by the Achilles, as multiple investigations have shown that failing to address this problem is the leading cause of surgical fixation failure.

The modified two-stage approach described in this report allowed for the reduction of extensive soft tissue swelling and prevented the progression of skin compromise before the final fixation procedure. Additionally, this method relieved tension on the Achilles tendon, allowed for stable fracture reduction, and repositioned the tendon to its natural anatomic position. This report highlights the value of a staged surgical approach utilizing the PARS technique and draws attention to the complex soft tissue concerns in the management of type II calcaneal tuberosity fractures. To assess complications, functional results, and the long-term durability of this surgical method in larger patient cohorts, further studies are necessary.
